# A spatial model of Wild Poliovirus Type 1 in Kano State, Nigeria: calibration and assessment of elimination probability

**DOI:** 10.1186/s12879-016-1817-3

**Published:** 2016-09-29

**Authors:** Kevin A. McCarthy, Guillaume Chabot-Couture, Faisal Shuaib

**Affiliations:** 1 Intellectual Ventures Laboratory, 3150 139th Ave SE, Bellevue, WA 98005 USA; 2 Federal Ministry of Health, Abuja, Nigeria

## Abstract

**Background:**

Since the launch of the Global Polio Eradication Initiative, all but three countries (Nigeria, Pakistan, and Afghanistan) have apparently interrupted all wild poliovirus (WPV) transmission, and only one of three wild serotypes has been reported globally since 2012. Countrywide supplemental immunization campaigns in Nigeria produced dramatic reduction in WPV Type 1 paralysis cases since 2010 compared to the 2000’s, and WPV1 has not been observed in Nigeria since July 24, 2014. This article presents the development and calibration of a spatial metapopulation model of wild poliovirus Type 1 transmission in Kano State, Nigeria, which was the location of the most recent WPV1 case and 5 out of 6 of the reported WPV1 paralytic cases in Nigeria in 2014.

**Methods:**

The model is calibrated to data on the case counts and age at onset of paralysis from 2003–2009. The features of the data drive model development from a simple susceptible-exposed-infective-recovered (SEIR) model to a spatial metapopulation model featuring seasonal forcing and age-dependent transmission. The calibrated parameter space is then resampled, projected forward, and compared to more recent case counts to estimate the probability that Type 1 poliovirus has been eliminated in Kano state.

**Results:**

The model indicates a 91 % probability that Type 1 poliovirus has been eliminated from Kano state as of October 2015. This probability rises to >99 % if no WPV1 paralysis cases are detected for another year. The other states in Nigeria have experienced even longer case-free periods (the only other state with a WPV1 case was Yobe, on April 19, 2014), and Nigeria is the last remaining country in Africa to experience endemic WPV1 transmission, so these results can be interpreted as an upper bound on the probability that WPV1 transmission is currently interrupted continent-wide.

**Conclusions:**

While the results indicate optimism that WPV1 transmission has been interrupted in Kano state, the model also assumes that frequent SIAs with high coverage continue to take place in Kano state through the end of the certification period. We conclude that though WPV1 appears to be on the brink of continent-wide elimination (WHO officially removed Nigeria from the list of polio-endemic countries on September 25, 2015), it is important for the polio program to maintain vigilance in surveillance and vaccination activities to prevent WPV1 resurgence through the WHO’s 3-year eradication certification period.

**Electronic supplementary material:**

The online version of this article (doi:10.1186/s12879-016-1817-3) contains supplementary material, which is available to authorized users.

## Background

In 1988, the Global Polio Eradication Initiative (GPEI) was launched, and since then, all but three countries (Nigeria, Pakistan, and Afghanistan) have interrupted endemic transmission of all three wild poliovirus (WPV) serotypes [1]. The world has been free of WPV serotype 2 (WPV2) since 1999 [2], and no cases of WPV3 have been reported since 2012 [1]. WPV1 transmission continues uninterrupted in Pakistan and Afghanistan, but the last case in Nigeria was observed on July 24, 2014 [3]. WPV1 paralysis case counts in Nigeria in 2010–2014 were substantially lower than during the 2000s, and this decline in cases has culminated in the announcement, on 25 September, 2015 [4], of the interruption of endemic WPV1 transmission in Nigeria.

This article describes a polio transmission model developed to investigate the interruption of WPV1 transmission in northern Nigeria. This question has been addressed [5–8] using other transmission models; of these, the models presented in [5, 8] are not calibrated specifically to the Nigerian context, and indicate that high (>95 %) confidence in WPV1 interruption may require case-free periods of 3 years or more, while [6, 7] present models specific to northern Nigeria, and find that as little as 1 year without a WPV1 paralysis case is sufficient to be confident that transmission has been interrupted. The model presented here is structurally distinct from the models of [6, 7], and provides further support for their conclusions that interruption can be confidently declared with a shorter case-free period. The model is calibrated to WPV1 case counts in Kano state, Nigeria. Historically, Kano state accounts for approximately 30 % of WPV1 paralytic cases in northern Nigeria (despite having only ~16 % of the total population), and 5 of the 6 cases reported in 2014 were from Kano state. The model is an individual-based model featuring generic susceptible-exposed-infective-recovered (SEIR) dynamics. The model was developed in a data-driven iterative fashion, beginning with a homogenously mixed, single-population SEIR model, to which age structure, seasonal forcing, and spatial transmission are progressively added to improve the ability of the model to reproduce the observed historical data.

## Methods

The generic disease branch of the individual-based disease modeling software EMOD DTK v1.6 was used to model polio transmission. The model was developed in an iterative fashion, beginning with a generic, individual-based SEIR model, with age structure, seasonal forcing, and division into spatial metapopulations progressively added to the model to improve fits to the data. Only children under 5 years of age are included in the model, as these children account for ~98 % of total WPV1 paralytic cases in the dataset for northern Nigeria. The 2006 Nigerian census reports that 1.8 million children under 5 years old lived in Kano state at that time [9]. The simulated birth rate maintains a population growth rate of 2.8 % [10]. Our simulated population consists of approximately 800 k agents in 2006 (averaged over simulation instances, as vital dynamics are stochastic in this model), so that each simulated individual represents approximately 2.2 real individuals. Simulation with 1.8 million agents resulted in unacceptably long instance runtimes. Reducing the simulated population prevented this issue from occurring, and as the model implements frequency-dependent transmission, the mean dynamics should not directly depend on the number of simulated agents, as long as that number is well above the critical community size.

Upon clearance of infection or a successful immunization with oral polio vaccine (OPV), individuals are modeled as being completely immune to future infection. Challenge studies have shown that individuals are in fact susceptible to reinfection. However, the probability of reinfection is strongly reduced after a successful infection or vaccination; the metastudy presented in [11] estimates that the relative odds of shedding serotype 1 poliovirus post-challenge among OPV-vaccinated individuals compared with unvaccinated individuals is 0.13. [12] presents an odds ratio of 0.15, with 80 % of unvaccinated individuals excreting virus post-challenge, compared with 37 % of OPV-vaccinated individuals. The OPV-vaccinated individuals in [12] were also observed to shed for a mean of 4.6 days, as opposed to 20.4 days among the unvaccinated, and the mean titer of virus excreted by OPV-vaccinated individuals was over 3 orders of magnitude lower than unvaccinated controls; naturally immune individuals exhibited similar characteristics to the OPV-vaccinated group. The reduced chance of infection, shorter shedding period, and reduced shedding titer of OPV- and WPV-experienced individuals combine to provide support to the simplifying assumption that the observed disease transmission dynamics are driven primarily by naïve individuals, rather than by reinfection of previously individuals.

The historical SIA campaign calendar for northern Nigeria (see Fig. 8) is implemented in simulation. In calibration of this model, the effects of SIA campaigns are parametrized by a campaign coverage multiplied by assumed vaccine take rates for different vaccine types. The product of the two represents the fraction of susceptible children immunized in a single SIA round and is the relevant quantity being calibrated; this product is constrained to lie between 0 and 1. Model calibration figures will present the campaign coverage alone, as the vaccine take rates are assumed to be constant. Trivalent OPV campaigns are modeled with a 15 % probability of successful vaccination against type 1, while monovalent OPV1 and bivalent OPV are modeled with a 20 % success probability each; these numbers are taken from 30-day seroconversion rates to a birth dose in India [13]. The efficacy of the multiple types of OPV has been measured in numerous studies [11, 13–22], and the results vary considerably across studies. The numbers employed here are on the lower end of observed single-dose take rates. Circulation of attenuated vaccine-derived virus is not modeled. Each simulation begins on Jan 1, 1985, with a 5-year period in which infected individuals are stochastically imported into the network. The mean importation rate is one infected individual per week per 10 k population for these 5 years, after which the disease is sufficiently established to survive without importation in the absence of vaccination. The SIA campaign calendar was provided by the Nigerian WHO and [23–26].

### Model development and calibration

The acute flaccid paralysis (AFP) surveillance database, maintained by the Nigerian WHO, provides the basis for model calibration; the model was calibrated only to data from 2003–2009. Only AFP cases with WPV1 shedding confirmed by the Global Polio Laboratory Network [27] are included. Incremental mixture importance sampling (IMIS) [28] was used for parameter space exploration and calibration. Though IMIS applies to deterministic models and true likelihood functions, the authors find it to work quite well at calibrating stochastic models given an objective function, so long as parameter-based variation in the objective function outweighs the stochastic variance in the objective function at a particular parameter set, as is the case for most of the calibrations presented below. The value of the objective functions evaluated at a particular parameter set and stochastic instance will be referred to as the score. The objective functions themselves are described in detail in Additional file 1. The data targets are the time series of case counts, binned twice per month; the distribution of age at onset of paralysis, binned into 6-month age bins; and the spatial distribution of cases at the LGA level.

The model was developed in an iterative fashion, beginning with a generic, individual-based SEIR model, and adding age structure, seasonal forcing, and division into spatial metapopulations progressively to improve fits and target additional features of the data. The model development and calibration section is split into subsections describing the reasoning and calibration at each step of development, beginning with a very simple (and poor) model and progressing through a couple of intermediate models before arriving at the finalized model. The reader may wish only to see the specification and calibration of the finalized model and results, contained in Additional file 1– Model Specification, Model Development – Spatial Model, and Results, respectively.

### Model development – Basic SEIR model

The incubation and infectious periods, base reproductive number, and the campaign coverage of a basic SEIR model were fit to these data. Campaign coverage is assumed to be constant over this time period, and campaigns are modeled as being randomly accessed by individuals, with no structured heterogeneity in individuals’ probabilities of being vaccinated in a single campaign. The infectious and incubation periods are of fixed duration, rather than exponentially distributed. The choice of a fixed duration maintains the benefit of having only a single parameter to calibrate for each, like the exponential distribution, while removing that distribution’s probability of individuals drawing unrealistically short infectious durations.

The case-to-infection ratio of WPV1 in immunologically naïve individuals is approximately 1:200 [29]. For the purposes of calibration, the modeled incidence over time is fit to the case count data using a Poisson approximation to a binomial objective function, which is maximized over an unconstrained case-to-infection ratio (for an investigation of overdispersion in the case counts relative to the model, and how this affects the results, please see Additional file 1, Overdispersion section). This construction provides a scale-independent fit of the shape of the modeled incidence curves to the case counts over time. In the high-incidence periods used for calibration and with a large simulated population, the average dynamics are quite insensitive to the total modeled population. More details about the objective functions employed are included in Additional file 1.

**Fig. 1:**
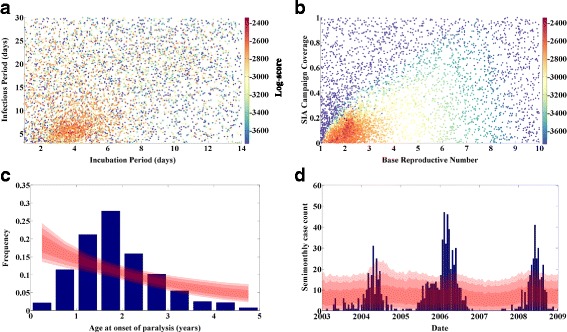
Calibration of basic SEIR model. **a** Log-score (color) vs. incubation and infectious period. **b** Log-score vs. R_0_ and campaign coverage. **c** Data (*blue*) and score-weighted distribution of simulations (68, 95, 99 % quantiles in *red*) for age distribution of paralysis cases. **d** Data (*blue*) and score-weighted distribution of simulations (68, 95, 99 % quantiles in *red*) for time series of case counts

Figure 1 presents the results of this calibration. Figure 1(a) and (b) show that the objective score is strongly dependent on R_0_ and SIA coverage, but relatively weakly dependent on the incubation and infectious periods; the space around 4 days exposed and 5 days infectious is preferred, but individual samples of comparably high score are present throughout the 2D space. Figure 1(c) and (d) present the target data as blue bars. The red contours indicate the 68 %/95 %/99 % quantiles of distributions constructed by weighting the outputs according to their score (were the objective functions true likelihoods, these would represent quantiles of the posterior distribution; as they are not, the statistical interpretation of these contours is not immediately obvious, but they serve to demonstrate how well the calibrated model reproduces the targeted features of the data). These figures demonstrate that the capability of this model to fit the data is rather poor; the age distribution of infection (as expected) is monotonically decreasing, in contrast to the data which peaks in the 1.5–2 year age bin. The simulated case counts over time do exhibit a low-amplitude oscillation with the 2-year periodicity exhibited by the data, but fail to reproduce the high amplitude of the outbreaks and long troughs of very low case counts in the data.

### Model development – Age dependent exposure and seasonality

The poor performance of a basic SEIR model is not unexpected. In a homogenously mixed SEIR model, new infections will be distributed uniformly among the susceptible population, the proportion of susceptibles will decline with age as older age groups have been exposed for longer, and the distribution of new infections will thus decline with age as well, as seen in the simulation results of Fig. 1(a). Similarly, oscillatory behavior in a homogenously-mixed SEIR models is expected to damp toward an equilibrium value; stochastic resonance can drive oscillations at the natural frequency of the system, but for the large population simulated here these oscillations are insufficiently large to explain the data. This model is presented primarily for comparison against more developed models, to demonstrate the value of additional data-driven complexity.

A number of mechanisms could potentially explain the discrepancy in the simulated age at infection distribution and the observed age at onset of paralysis. Age-dependence in the case-to-infection ratio has been reported in the past [30]; however, the measurements indicate no more than a factor of 2 difference over the first 5 years of life, insufficient to transform the simulated distribution into the data. Maternal antibodies are another obvious potential explanation, but previous work indicates that these wane with a half-life on the order of 3–4 weeks [31–35], much too rapidly to solely explain a rising attack rate over the first 2 years of life. The hypothesis implemented into the model is reduced mixing by young children, such that the force of infection is reduced by an age-dependent factor. For simplicity’s sake, this age-dependent scaling factor is assumed to be multiplicative, linearly dependent on age, and bounded between 0 and 1 (where 0 represents no exposure to infectivity, and 1 represents full exposure. To clarify, let *β*(*a*, *t*) represent the force of infection from all infectives onto susceptibles of age *a*, then:

$$ \beta \left(a,\ t\right) = \left\{\begin{array}{ll}\kern2.5em 0,\hfill & {f}_0+{f}_1a\le 0\hfill \\ {}{\beta}_0\left({f}_0+{f}_1a\right),\hfill & 0 < {f}_0+{f}_1a<1\hfill \\ {}\kern2.25em {\beta}_0,\hfill & {f}_0+{f}_1a\ge 1\hfill \end{array}\right. $$

The model is recalibrated, with the slope and age-0 intercept of this scaling factor included as additional dimensions in the calibration.

**Fig. 2:**
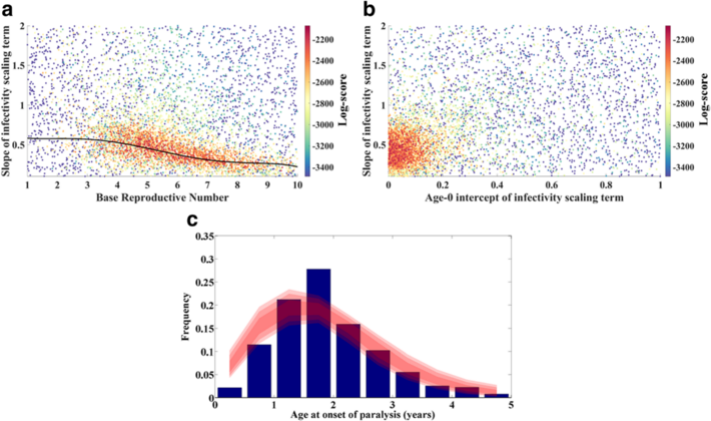
Calibration of SEIR model w age-dependent transmission. **a** Log-score (color) vs. base infectivity and the slope of age-dependent susceptibility in early life (described in text). **b** Log-score (color) vs. the age-0 intercept and slope of age-dependent susceptibility in early life. **c** Data (blue) and score-weighted distribution of simulations (68, 95, 99 % quantiles in red) for age distribution of paralysis cases

Figure 2 shows the results of this calibration. The simulated age at infection distribution depends most strongly on the base reproductive number and the slope and intercept of the age-dependent mixing coefficient. Figure 2 only presents the score in the 2D space of R_0_ and the slope of the age-dependent mixing; the most likely points for the intercept are clustered near 0, as expected due to the very low case counts in the 0–6 month age bin. It should be noted that this reduced mixing induces a shift in the true R_0_ that depends on the age-dependent mixing factor and the age distribution of the population; for the remainder of this paper, R_0_ (or base reproductive number in figure axes) will refer to the daily force of infection times the infectious period, which represents the R_0_ within a fully mixed population.

For subsequent calibrations and model runs, the intercept of age-dependent mixing will be fixed at 0 (see Fig. 2 panel b), and the relationship between the slope and the base reproductive number will used to collapse these two onto a single dimension; that is, R_0_ will remain a freely fitted parameter, but at a given R_0_, the age-dependent mixing slope is determined by the fitted curve shown in Fig. 2a.

The next model iteration aims to address the strong periodicity and high amplitude of WPV1 outbreaks, by adding a seasonal forcing term to the infectivity:

$$ \beta \to \beta \left(1+A\left( \cos \left(\frac{t-{t}_0}{365}\right)\right)\right) $$

Seasonal outbreaks have been observed in many infectious diseases [36–43], and seasonally-forced models have been shown to exhibit bifurcations from annual outbreaks to dynamics with longer periodicities [44, 45]. The 2-year periodicity of WPV1 outbreaks in Kano state constrains the space of combinations of base reproductive number, SIA campaign coverage, and seasonal forcing amplitude.

**Fig. 3:**
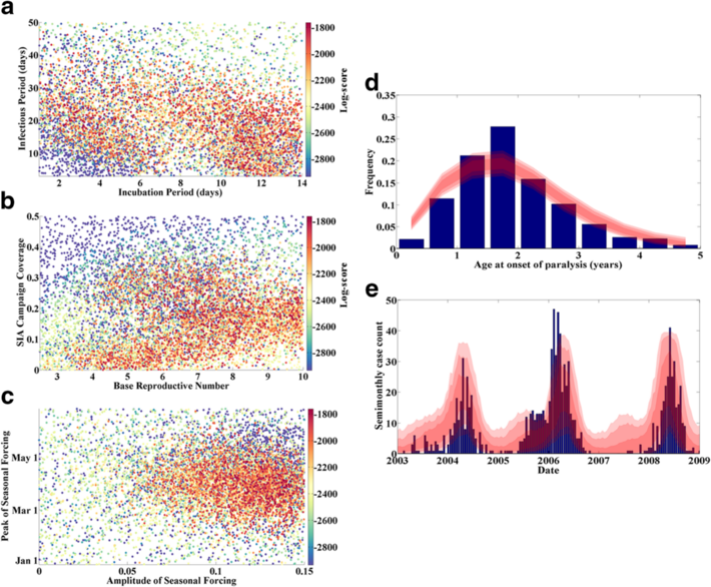
Calibration of SEIR model w age-dependent transmission and seasonal forcing. **a** Log-score (color) vs. incubation and infectious periods. **b** Log-score vs. R_0_ and campaign coverage. **c** Log-score (color) vs. the phase and amplitude of seasonal forcing in infectivity. **d** Data (blue) and score-weighted distribution of simulations (68, 95, 99 % quantiles in red) for age distribution of paralysis cases. **e** Data (blue) and score-weighted distribution of simulations (68, 95, 99 % quantiles in red) for time series of case counts

Figure 3 shows the results of this calibration procedure. The fit to the case count data is substantially improved by the addition of seasonal forcing to the model, and the fit to the age distribution remains good. The overall peak score has increased dramatically; compare the color bar limits between Fig. 3 and Fig. 1. The structure of the objective function presents some interesting features, with multiple broad regions of high score. The incubation period and infectious period are not strongly constrained by the fits, but existing studies indicate a very short delay from exposure to the onset of viral excretion [46, 47]. When the calibration space is restricted to include only incubation periods between 1 and 5 days, the score-weighted mean infectious period is 27 days, a value which is also within the range supported by shedding studies [12, 46, 48]. Inclusion of these features appears to have improved the fit of these biological parameters relative to the SEIR model without these additional features, which inferred a mean infectious period of only a few days, Fig. 1. The seasonal forcing model is best fit with an amplitude of 0.11 and a peak on April 10. These values will be fixed in future calibration runs, while R_0_ and the mean SIA campaign coverage remain as calibration parameters.

As noted early in this section, the time series of cases from data and infections from simulation are compared using a scale-independent fit, with the case-to-infection ratio representing a free scaling parameter in the fit. While this simple SEIR model with minimal added complexity is able to reproduce the outbreak dynamics, the weighted mean case-to-infection ratio is about 1:1650, much lower than the expected 1:200 ratio for WPV1. Assuming the expected 1:200 ratio, the model overpredicts the observed number of cases by a factor of approximately 8 on average. This discrepancy may indicate the existence of some unmodeled complexities. Multiple hypotheses could all plausibly contribute to this discrepancy: The assumption of complete immunity after infection with OPV or WPV rather than the biologically justifiable partial immunity and waning immunity could allow for some degree of transmission to proceed through individuals who are less likely to present paralysis (though for reasons given previously, the authors expect this contribution to transmission to be small), individual-level heterogeneity in transmission (through host factors or disparate social networks), and the treatment of Kano as a single mixing population, rather than accounting for the spatial distribution of the population and how this affects transmission. The model development proceeds by investigating this last hypothesis, though all are likely to contribute.

### Model development – Spatial model

The polio case dataset used for calibration reports cases at the scale of local government areas (LGAs), providing spatial information that may also be incorporated into model calibration, which requires splitting the single-population model presented above into a set of metapopulations. Construction of the spatial metapopulation model is covered in detail in Additional file 1. WorldPop [49, 50] population maps are the basis for metapopulation construction. Spatially contiguous features of high population density are identified and aggregated into population centers, with the total population placed at the population-weighted mean latitude and longitude. Any remaining low-density background population is aggregated to the nearest population center. Nodes with total populations below 1000 individuals are merged with their nearest neighbor. This process results in 1322 nodes. Each of these population centers is a separate metapopulation, or node, in the model.

Nodes are internally well-mixed (with the age-dependent mixing described above), and coupled to each other through migration of individuals. Individuals are assumed to take only round-trip migration, with an average duration of 1 day at the destination node. Individual migration rates are assumed to follow a gravity-like model, with the rate linearly increasing with destination population, inversely linear with the distance to the destination node, and independent of the population of the home node.

$$ {R}_{ij}=A*\frac{p_j}{d_{ij}} $$

The migration rate is implemented in units of per-individual per day, so that the total flow from node *i* to node *j* is implicitly linear in the population of node *i.* The network is not completely connected; migration from a node only occurs to its 8 nearest nodes and its 30 remaining most probable destinations.

Calibration of the spatial model covers a 5-dimensional parameter space including the coverages of routine immunization (RI) and campaign immunization, the base reproductive number, a coefficient of variation in campaign coverage (though not RI) at the LGA level, and the scaling constant of the overall rate of migration.

The spatial parameters added to the model necessitate the addition of components to the objective function that are sensitive to spatial effects. The new target data include the number of LGAs reporting at least one case within a 3 month time window, and the annual case counts in each of the 44 LGAs of Kano state. The objective functions do not aim to match specific LGAs between simulation and data, but target the general heterogeneity in LGA-level case counts.

**Fig. 4:**
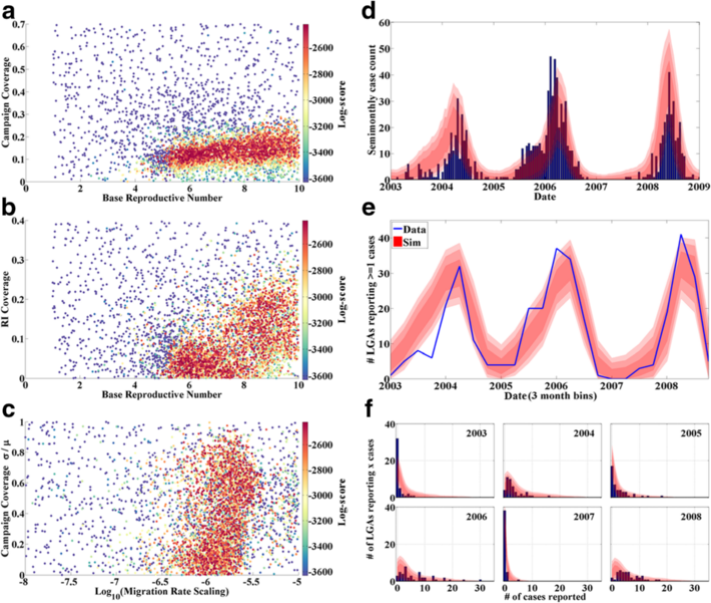
Calibration of the spatial polio-like model. **a** Log-score (color) vs. R_0_ and campaign coverage. **b** Log-score (color) vs. R_0_ and RI coverage. **c** Log-score (color) vs. scaling term in the migration model (x) and LGA-level coefficient of variation in campaign coverage. **d** Data (blue) and score-weighted distribution of simulations (68, 95, 99 % quantiles in red) for time series of case counts. **e** Data (blue) and score-weighted distribution of simulations (68, 95, 99 % quantiles in red) for the number of LGAs reporting at least 1 case in a given time period. **f** Data (blue) and score-weighted distribution of simulations (68, 95, 99 % quantiles in red) for distribution of annual case counts among the 44 LGAs

**Fig. 5:**
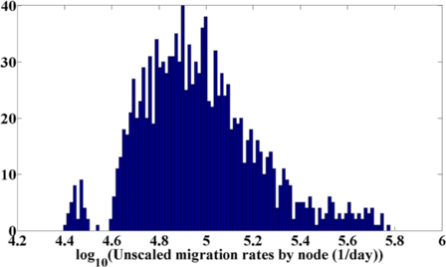
Histogram of unscaled outbound migration rates by node. The high-score region shown in Fig. 4 centers around a scaling factor of -6, indicating expected migration rates for most nodes at ~0.1 per person per day

Results of the calibration are presented in Fig. 4 (except for the age of infection distribution, suppressed as the fit is of similar quality to those presented previously). Panels a-c present the log-score distributions in 2D planes similar to those shown previously. The best-fit campaign and RI coverages both increase with infectivity, as expected. The LGA-level coefficient of variation of the campaign coverage is not strongly constrained, though the scaling factor on the migration rate is constrained to lie within approximately 1 order of magnitude. To provide an interpretation for this result, Fig. 5 presents a histogram of the unscaled migration rates by node. Scaling this histogram by the high-score region in Fig. 4(c) reveals that the best fits occur when individuals in most nodes have outbound travel frequencies from about once per 3 days to once per 30 days.

The inferred case-to-infection ratio from the metapopulation model was approximately 1:1200. While still much higher than the expected 1:200, this does represent an improvement in the overall scale of the simulated outbreaks when compared with the single-population model. Other unmodeled heterogeneities may be responsible for the continuing discrepancy; perhaps the modeled population may be viewed as an undervaccinated subpopulation or a subpopulation at high risk relative to the total population.

After calibration of the spatial model, the model is applied to the question of elimination. At the time of writing, no cases of WPV1 paralysis have been observed in Nigeria since July 24, 2014. The WHO standard for certifying interruption of WPV in a region is 3 years without an observed paralysis case. Other models have been applied to the question of elimination probability given a case-free period [5–8, 51], and this model is used to address the same question.

The calibration parameter space from the 2003–2009 calibration is resampled for each simulation, with the priors constricted to reflect the regions of high score in Fig. 4. At this stage, the fitting timeframe is extended to include all data until the most recently observed WPV1 case, and the simulation timeframe is extended to 2020. Importation pressure from other states in northern Nigeria is added to the model because Kano state underwent a period of 20 months in 2009–2010 without a single WPV1-positive paralysis case. This is a low-probability event in this model and indicates the possibility of a local elimination followed by reimportation.

Increasing campaign coverage over time is also added to the model for this investigation, as the available data on campaign coverage in Kano indicates that campaign coverage improved in the late 2000s and into the 2010s (lot quality assurance sampling (LQAS) data, provided by the Nigerian WHO). In this exploration, campaign coverage is assumed to increase linearly with time, beginning at some time between Jan 1, 2005 and Jan 1, 2010. After the last campaign in the historical and planned campaign calendar (Dec. 2015), campaigns are assumed to use bOPV or mOPV1, with campaigns occurring at a constant rate of 9/year.

Finally, the scale-free fitting utilized in calibration is not appropriate for addressing the question of pathogen elimination, as the size of the mixing population and the case-to-infection ratio affect how long infection can silently transmit. Therefore, the simulated population is reduced to match the scale of the simulated infections with the observed paralysis data at a case-to-infection ratio of 1:200. The final simulated population is ~284000 in Mar 2006, compared to the census population of 0–5 year olds of 1.8 million in the same year. Figure 7 demonstrates that the quality of the calibration fits remains comparable to the larger population simulations.

## Results and discussion

The probability that WPV1 has been eradicated at day *t* (i.e., the number of infected *I(t)* = 0 at day *t*), given that the cumulative number of cases observed *N*_*c*_*(t)* since the last case is 0, is:

$$ {P}_{erad}(t)=P\left(I(t)=0\Big|{N}_c(t)=0\right) = \frac{P\left({N}_c(t)=0\ \Big|I(t)=0\right)\ P\left(I(t)=0\right)}{P\left({N}_c(t)=0\right)} $$

The probability of observing 0 cumulative cases is a binomial draw from the cumulative number of infections *I*_*c*_*(t),* and *P(I(t) = 0)* is an indicator function that is either 0 or 1 for each simulation and time point.

$$ {P}_{erad}(t) = \frac{P\left({N}_c(t)=0\ \Big|I(t)=0\right)}{P\left({N}_c(t)=0\right)} = \frac{{\displaystyle {\sum}_{\left\{ sims\ \Big|\ I(t)=0\right\}}}\  Bin\left(0\Big|{I}_c(t),\ .005\right)}{{\displaystyle {\sum}_{\left\{ all\  sims\right\}}} Bin\left(0\Big|{I}_c(t),\ .005\right)} $$

**Fig. 6:**
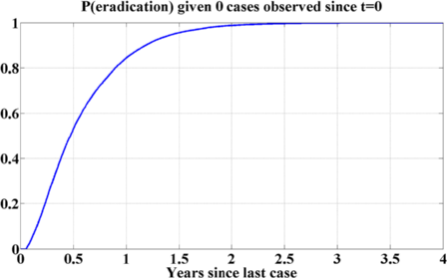
Estimated probability of WPV1 elimination given 0 cases observed since the last confirmed WPV1 case (Jul 24, 2014 in Kano state)

A total of 16,350 simulations were performed, of which 8113 survive until the time of the most recently observed case. Figure 6 presents the probability of elimination vs. time since the last case. One year out from the last case, this model predicts a 91 % probability of elimination, which rises to nearly 99 % 2 years out.

**Fig. 7:**
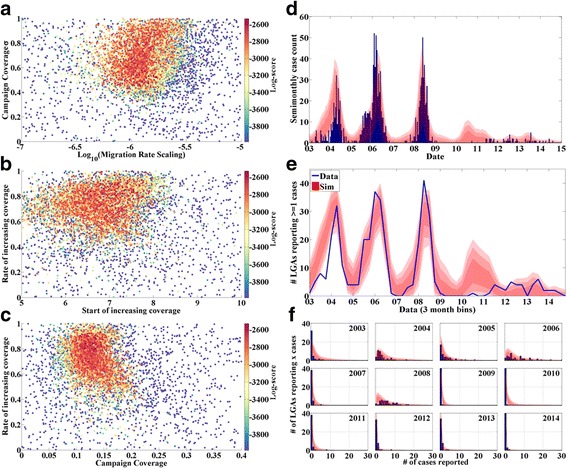
Results of date of elimination simulations. **a** Log-score vs. the scaling factor in the migration rate model and the LGA-level coefficient of variation in campaign coverage. **b** Log-score vs. the time at which campaign coverage begins to increase, and the relative annual rate of increase. **c** Log-score vs. the base campaign and the relative annual rate of coverage increase. **d** Data (blue) and score-weighted distribution of simulations (68, 95, 99 % quantiles in red) for age distribution of paralysis cases. **e** Data (blue) and score-weighted distribution of simulations (68, 95, 99 % quantiles in red) for time series of case counts. **f** Data (blue) and score-weighted distribution of simulations (68, 95, 99 % quantiles in red) for the number of LGAs reporting at least 1 case in a given time period

Figure 7 presents a selection of slices in the score space and comparisons of simulations to data; Fig. 7(a) presents the log-score in the space of the migration rate scaling and the coefficient of variation of campaign coverage. A comparison with Fig. 4 reveals that including the years from 2009–2014 and the rise in campaign coverage over time allows for stronger constraints on this heterogeneity; the most likely values are quite high, which allows local eliminations in the most well covered LGAs but continued circulation in the set of poorly covered LGAs. The model fits best when campaign coverage begins to increase sometime between 2006 and 2008, with quite rapid increases of 50–100 % per year. The increase in coverage, as implemented, is relative to the “base” coverage, which ranges from 10–15 %. The best-fit absolute rates of coverage increase can be estimated from Fig. 7(c) and range from about 5 to 15 % additional absolute coverage (vaccine efficacy remains constant) per year.

**Fig. 8:**
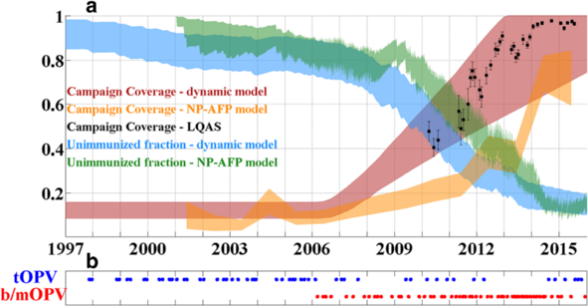
**a** 95 % quantiles of the score-weighted distributions of (*red*) statewide mean SIA coverage over time as estimated in calibration of the presented model; (*orange*) statewide mean SIA coverage over time as estimated using a model based on non-polio acute flaccid paralysis (NP-AFP); (*black*) statewide mean SIA coverage as estimated from lot quality assurance sampling (data provided by Nigerian WHO); (*blue*) statewide mean vaccine-derived immunity in 0–5 year olds from the presented model; (*green*) statewide mean vaccine-derived immunity in 0–5 year olds from a statistical model deriving campaign coverage from the reported vaccine dose histories of non-polio acute flaccid paralysis cases. The NP-AFP immunity model is an extension of that presented in [56] (publication detailing newer model is in preparation). **b** Illustration of historical campaign calendar in Kano state

Figure 8 provides a more detailed illustration of the fraction of susceptibles who are successfully immunized in each SIA round over time. The model predicts quite low values of immunization within this population throughout the early-mid 2000s. These rates are sufficient to drive the system into sharp peaks of 2-year periodicity, but the calibrated immunization rate rises rapidly through the late 2000s to match the much lower case counts of 2010–2015. A portion of the posterior distribution of the statewide rate exceeds 20 % beginning in 2014, indicating that the chosen values for take rate may have been too low (as campaign coverage should not exceed 100 %).

A number of features of this model may contribute to an overall optimistic picture of elimination, however. The campaign coverage is assumed to have been rising over time for approximately the past 10 years, which allows the model to fit the dramatically reduced incidence from 2010–2015 compared to the previous decade, and this increasing coverage is assumed to continue into the future. Furthermore, future modeled campaigns were assumed to be mOPV1 or bOPV, but as the WPV1 case-free period grows, the use of tOPV, which is less effective at providing immunity against Type 1 [13], has increased correspondingly as the focus shifts to eliminating circulating vaccine-derived poliovirus Type 2 (cVDPV2). The closed nature of the modeled population also does not admit the possibility of exportation to at-risk populations connected to that of Kano state; even if transmission is currently declining towards elimination, exportation to outside populations could provide a reservoir for re-establishment. Time-varying or LGA-specific levels of routine immunization are not accounted for in this model; nor is the co-administration of inactivated polio vaccine (IPV) with OPV.

The probability of polio elimination given a case-free period has been addressed in previous work, in other contexts [8, 51] and in Nigeria [5–7]. The results of this model are similar to other recently published results considering the Nigerian context, which are derived from models differing widely in structure from the present model. Of these models, the present model uniquely features spatially structured metapopulations with heterogeneous immunization coverage, which admits certain phenomena of interest in the near-elimination regime – localization of transmission chains and the possibility of escape from an infected region to another region that is not experiencing local transmission. The calibration procedure presented is also unique among the Nigeria models in methodology and in the data targets. The confluence of results from multiple models provides some confidence that the optimistic biases discussed above do not drive this result, and provide support to the WHO’s recent removal of Nigeria from the list of polio-endemic countries [4].

## Conclusions

The model predicts that at the time of writing, October 2015, the lack of any cases since July 24, 2014 [3] indicates a probability of 91 % that WPV1 has been eliminated from Kano state. The other states in Nigeria have experienced even longer case-free periods (the only other state with a WPV1 case in 2014 was Yobe, on April 19, 2014), and as Nigeria is the last remaining country in Africa to experience endemic transmission of WPV1, this probability can be interpreted as an upper bound on the probability that endemic transmission of WPV1 has been interrupted continent-wide. If another year passes without any observed WPV1 paralysis cases, this model indicates a 99 % chance of elimination.

The model assumes that frequent SIAs with high coverage continue to take place in Kano state through the end of the simulation period, and though these results are optimistic that WPV1 transmission in Kano has been or will soon be interrupted, these results do not provide support for any reduction in vaccination activities in the immediate future. The results indicate that it is important for the polio program to maintain vigilance in surveillance and vaccination activities to prevent WPV1 resurgence through the WHO’s 3-year eradication certification period.

**Added June 2016:** On 23 March 2016, a cVDPV2-positive environmental sample was isolated in the Maiduguri district of Borno state, Nigeria, and determined to be genetically linked to a strain last observed in May 2014 [52]. As calibration of the presented model focused exclusively on WPV1, it cannot be directly applied to silent circulation of cVDPV2; investigations of silent cVDPV2 circulation can be found in [6, 7]. Both references find that long-term silent circulation of Type 2 is more likely than Type 1, even with perfect AFP surveillance. The difference in case-to-infection ratios (approximately 1:200 for Type 1, 1:1900 for Type 2 [29]) likely accounts for much of the difference in silent circulation of the two serotypes, but the appearance of this environmental sample demonstrates that silent circulation remains a major concern in certifying global poliovirus eradication, particularly after the April 2016 worldwide withdrawal of OPV2 [53].

**Added August 2016:** Since preparation of this manuscript, multiple persons infected with WPV1 were discovered in areas of Borno state previously inaccessible to programmatic activities due to violence and instability. One WPV1-positive paralysis case was found along with multiple WPV1-positive healthy close contacts, and a second paralysis case that was WPV1-negative was found to have a WPV1-positive close contact [54]. Genetic sequencing indicates a period of approximately 5 years between the virus causing these infections and the most recent known related infection [55]. These observations of WPV1 follow a recent discovery of a cVDPV2 circulating silently for nearly 2 years. These discoveries emphasize the necessity of high-quality surveillance during the polio endgame and certification process. Many other regions of Borno remain inaccessible to surveillance, limiting knowledge about the extent of the WPV1 transmission within the state.

This approximately 5 years of silent circulation may call into question the presented results. The model’s predicted probability of elimination vs. time since the last paralytic case depends on two crucial assumptions: that SIAs continue to take place after the last observed paralysis case, and that surveillance for new paralytic cases is of high quality. Neither of these assumptions hold in the inaccessible areas of Borno. This and other models approaching the question all find 5 years of circulation without generating a new paralysis case extremely unlikely [5–8], indicating a probable surveillance failure throughout the inaccessible regions of Borno. These cases, along with the cVDPV2 previously observed in Borno, highlight the existential risk that inaccessible populations present to polio eradication.

## Additional file

Additional file 1: Includes descriptions of model specification, definitions of the objective functions in calibration, investigations of overdispersion, and construction of the metapopulation model. (DOCX 2691 kb)

## Abbreviations

AFP: Acute flaccid paralysis
bOPV: Bivalent oral polio vaccine
GPEI: Global polio eradication initiative
IMIS: Incremental mixture importance sampling
IPV: Inactivated polio vaccine
LGA: Local governmental area
mOPV#: Monovalent oral polio vaccine type #
OPV: Oral polio vaccine
RI: Routine immunization
SEIR: Susceptible-exposed-infective-recovered
SIA: Supplemental immunization activity
tOPV: Trivalent oral polio vaccine
WHO: World health organization
WPV#: Wild polio virus type # (1/2/3).
