# Supplementary material for: A spatial model of Wild Poliovirus Type 1 in Kano State, Nigeria: calibration and assessment of elimination probability
Source: BMC Infect Dis. 2016 Sep 29;16:521. doi: 10.1186/s12879-016-1817-3 (PMC5041410; doi:10.1186/s12879-016-1817-3)
Supplement: Additional file 1: — Includes descriptions of model specification, definitions of the objective functions in calibration, investigations of overdispersion, and construction of the metapopulation model. (DOCX 2691 kb) [file 12879_2016_1817_MOESM1_ESM.docx]

**Supplemental Materials**

**Model Specification**

The EMOD model is an individual-based stochastic model of disease transmission, with support for campaign implementation, heterogeneous transmission, and spatially segregated populations coupled by individual-level migration; the software also includes sub-packages implementing “generic”, vector-borne, water-borne, airborne, and sexual disease transmission. The EMOD software is available for download at <http://idmod.org/idmdoc/#EMOD/EMODBuildAndRegression/BuildingEMODTOC.htm>. The model presented in this manuscript was developed within the EMOD “generic” simulation framework, implementing a discrete-time, individual-based form of the well-known SEIR (susceptible-exposed-infectious-recovered) model. Below, we review the adjustments to the simple SEIR model that were implemented in this model, specify the model in terms of the individual-level state transition dynamics, and specify the population-level model as discrete-time differential equations with stochastic terms.

A number of adjustments to the basic SEIR model assumptions were made in developing the model presented in this manuscript. The infectious and incubation periods (γ and σ, respectively) were modeled using constant times spent in a compartment, rather than the exponential distribution implied by the rate parameters in an SEIR model, changing the parameters from rates to delays in the equations. The mortality rate ν is implemented as a function of age ν(*a*), with values obtained from DHS[1]. Annual forcing in the transmission parameter β is also implemented to reproduce the seasonal outbreak structure in the case count data, making β a function of time.

The model includes a linear rising exposure to infectiousness with age, which provides a reasonable fit to the data and presents fewer terms for inference than an age-stratified who-acquires-infection-from-whom (WAIFW) matrix. In terms of model specification, β becomes a function of age as well as time.

| $\beta\left( a, t \right)= \left\{ \begin{matrix} 0 , f_{0}+f_{1}a\leq0 \\ \beta_{0}\left( f_{0}+f{}_{1}a \right)\left[ 1+A\cos\left( \frac{t-t_{0}}{365} \right) \right], 0< f_{0}+f_{1}a<1 \\ \beta_{0}\left[ 1+A\cos\left( \frac{t-t_{0}}{365} \right) \right], f_{0}+f_{1}a\geq1 \end{matrix} \right.$ | ( 1 ) |
| --- | --- |

Where $\beta\left( a, t \right)$ represents the force of infection from all infectives onto susceptibles of age *a*. Routine immunization is modeled as a probabilistic transition from the S class to the R class in the first timestep of life, and campaign immunization is treated as a probabilistic transition from S to R in the timestep in which the campaign takes place.

Kano state is divided into metapopulations Individuals of any disease state and age migrate between metapopulations (nodes) according to the procedure described in the supplemental section Metapopulation Model Construction. The infection process in a given node and at a given timestep is governed by individuals in that node at that timestep only. Individuals migrate between nodes (from a home node *i* to destination node *j*) with relative destination node rates according to a gravity-like model of migration; migration is modeled as one-day round-trips to prevent unrealistic population accumulation in the largest nodes.

| $M{}_{ij}={\alpha p}_{j}/d_{ij}$ | ( 2 ) |
| --- | --- |

**Individual-level transitions**

With all of this in hand, individuals can be specified by a state space of {disease state *X*, age *a*, time in disease state *t_X_,* home node *i*, current node *j*}. The absolute simulation time *t* also plays a role due to immunization campaigns on specific dates and seasonal dynamics; this simulation state variable is shared by all individuals. Disease state transitions, vital dynamic transitions, and migration transitions are treated independently from one another, simplifying the specification of the transition space. The individual-level state transitions are presented below in the order in which they are processed in simulation.

The first transition in a given timestep is aging:

| $p(\left\{ X, a, t_{X}, i, j \right\}\to\{X, a+\Delta, t_{X}+\Delta, i, j\})=1$ | ( 3 ) |
| --- | --- |

Next, immunization interventions are processed:

| Routine Immunization | $p\left( \left\{ S, 0, 0, i, i \right\}\to\left\{ R, 0, 0, i, i \right\} \right)=C_{RI}$ | ( 4 ) |
| --- | --- | --- |
| Campaign Immunization | $p\left( \left\{ S, a, t_{S}, i, j \right\}\to\left\{ R, a, 0, i, j \right\} \right)=C_{kj}\delta(t-t_{k})$ | ( 5 ) |

Where $C_{RI}$ indicates the coverage of routine immunization, $C_{kj}$ indicates the effective coverage of campaign *k* in node *j*, $\delta$ is the Kronecker delta function, and $t_{k}$ is the date of campaign *k*.

The infectious dynamics follow:

|  | $p\left( \left\{ S, a, t_{S}, i, j \right\}\to\left\{ E, a, 0, i, j \right\} \right)=1-exp\left( -\frac{\Delta\beta\left( a, t \right)I_{j}\left( t \right)}{N_{j}\left( t \right)} \right)$ | ( 6 ) |
| --- | --- | --- |
|  | $p\left( \left\{ E, a, t_{E}, i, j \right\}\to\left\{ I, a, 0, i, j \right\} \right)=\delta(t_{E}-\sigma)$ | ( 7 ) |
|  | $p\left( \left\{ I, a, t_{I}, i, j \right\}\to\left\{ R, a, 0, i, j \right\} \right)=\delta(t_{I}-\gamma)$ | ( 8 ) |

*S, E, I, R* without subscripts indicate individual-level disease states, and with subscripts *j* indicate the total population in state *X* in node *j*. *N­_j_* indicates the total population of node *j*.

Deaths are processed next:

| $p(\left\{ X, a, t_{X}, i, j \right\}\to\{\emptyset\})=\Delta\nu(a)$ | ( 9 ) |
| --- | --- |

Followed by migration – again, outbound migration follows the rates in Eq. (ref), homebound migration always takes place the following day:

| $p(\left\{ X, a, t_{X}, i, i \right\}\to\{X, a, t_{X}, i, j\neq i\})={\Delta M}_{ij}$ | ( 10 ) |
| --- | --- |
| $p\left( \left\{ X, a, t_{X}, i, j\neq i \right\}\to\left\{ X, a, t_{X}, i, i \right\} \right)=1$ | ( 11 ) |

Finally, new births are processed:

| $N(\left\{ \emptyset\right\}\to\left\{ S, 0, 0, i, i \right\})=Poiss(\Delta\mu N_{i}\left( t \right))$ | ( 12 ) |
| --- | --- |

Equations ( 1-12 ) combine to specify the agent-based model as implemented. This specification can also be converted into a set of stochastic difference equations; the resulting equations are rather unwieldy and somewhat difficult to read, limiting how informative they are to the reader. As a compromise, equations ( 12-17 ) describe the corresponding deterministic difference equations, which are more immediately interpretable. Stochasticity would be implemented by a conversion of each of the appropriate terms into random draws from distributions at each timestep (ensuring that matched terms, e.g., the loss of susceptibles to infection and gain of new infectives, or the round-trip migration terms, are drawn only once rather than twice).

| $S_{i}\left( a+\Delta, t+\Delta\right)=\mu\left( 1-C{}_{RI} \right)\Delta N_{i}\left( t \right)\delta\left( a \right)+S_{i}\left( a, t \right)\left[ 1-\nu\left( a \right)\Delta-C_{ki}\delta\left( t-t_{k} \right)-\frac{\beta\left( a, t \right)\Delta I_{i}\left( t \right)}{N_{i}\left( t \right)} \right]+M_{i}\left( S \right)$ | ( 13 ) |
| --- | --- |
| $E_{i}\left( a+\Delta, t+ \Delta\right)=E_{i}\left( a,t \right)\left[ 1- \nu\left( a \right)\Delta\right]+\frac{\beta\left( a, t \right)\Delta S_{i}\left( a,t \right)I_{i}(t)}{N_{i}\left( t \right)}-E_{i}\left( a,t-\sigma\right)+M_{i}(E)$ | ( 14 ) |
| $I_{i}\left( a+\Delta, t+\Delta\right)=I_{i}\left( a, t \right)\left[ 1- \nu\left( a \right)\Delta\right]+E_{i}\left( t-\sigma\right)-I_{i}\left( t-\gamma\right)+M_{i}(I)$ | ( 15 ) |
| $R_{i}\left( a+\Delta, t+\Delta\right)=R_{i}\left( a, t \right)\left[ 1- \nu\left( a \right)\Delta\right]+I_{i}\left( t-\gamma\right)+M_{i}\left( R \right)+C_{k}\delta\left( t-t_{k} \right)S_{i}\left( a, t \right)+\mu C_{RI}\Delta N_{i}\left( t \right)\delta\left( a \right)$ | ( 16 ) |
| For each class *X*, node *i,* $M_{i}\left( X \right)=\sum_{j} {[M}_{ji}X_{j}\left( a, t \right)-M_{ij}X_{i}\left( a, t \right)+M_{ij}X_{i}\left( a-\Delta, t-\Delta\right)-M_{ji}X_{j}\left( a-\Delta, t-\Delta\right)]$ | ( 17 ) |
|  |  |

Where $\delta\left( a \right)$ represents a delta function indicating that all new births enter at age 0, the delay terms $\Gamma$ and $\psi$ are defined as multiples of$\Delta$, the term $M_{i}(X)$indicates the balance of migration of individuals of class X into and out of node i (the last two terms in this equation balance the round-trip migration implemented in this model - the individuals who entered/left the node in the last step must return home in this step), $M_{ij}$indicates the per-capita rate of migration from node i to node j*,* $C{}_{RI}$ indicates the coverage of routine immunization (assumed to take place at birth), $C_{k}$ and $t_{k}$ indicate the effective coverage (coverage times vaccine efficacy) and date, respectively, of campaign k, and omission of the age *a* (e.g., $I_{i}\left( t \right)$) indicates summation over all ages.

As noted in the model development section, not all parameters of the model are jointly estimated; rather, subsets of the parameter space are estimated in a sequential fashion. This approach is chosen to avoid the “curse of dimensionality”, as the computational cost of evaluating the model is somewhat high (order 2-6 hours per instance, depending on the parameter values).

**Objective functions**

A Poisson-approximated binomial objective function was used to compare the time series of case count data $C_{t}$ with the simulated time series of infections $I{}_{t}$. Total cases from data and total infections from simulation are both binned into 2-week intervals, and the simulation score is the product over time bins of these binomial objective functions. To remove any dependence on the total simulated population size, the objective function is maximized over potential values of the case-to-infection ratio (which serves as the binomial probability parameter, and is independent of the time bin. This choice allows for fitting the shape of the trajectory with a degree of freedom in the scale).

|  | $\mathcal{L}\left( \vec{\theta} \right\vert C_{t})= \max_{A} \prod_{t} Poiss(C_{t}\vert I_{t}*A)$ | ( 18 ) |
| --- | --- | --- |

The value of *A* that maximizes this objective is employed as the unique value of the case-to-infection ratio in the remainder of the components of the objective function for that particular simulation.

A Dirichlet-multinomial posterior predictive distribution was used as the objective function to compare the age distribution of cases in data and sim. For each simulated parameter set $\vec{\theta}$, the ages of all individuals who become infected are recorded, binned into 10 6-month age bins, and downsampled by the case-to-infection ratio to provide an expected vector of simulated case counts by age, $\vec{k_{s}}$. The age-binned case counts in data, $\vec{k_{d}}$, are assumed to follow a multinomial distribution with probability vector $\vec{p}$. $\vec{p}$ itself is assumed to follow a Dirichlet distribution, which is informed by the simulation results by updating a symmetric Dirichlet prior with concentration parameter 1 to a posterior that accounts for the “observations” from simulation:

|  | $Dir\left( \vec{p} \vert\vec{1} \right) \underset{\to}{} Dir\left( \vec{p} \vert\vec{1}+ \vec{k_{s}} \right)$ | ( 19 ) |
| --- | --- | --- |

The score of $\vec{\theta}$ is then approximated using the posterior predictive distribution computed by marginalizing over $\vec{p}$:

|  | $\mathcal{L}\left( \vec{\theta} \right\vert d)= P\left( d \vert\vec{\theta} \right)= \int P\left( d \vert\vec{p} \right)P\left( \vec{p} \vert\vec{\theta} \right)d\vec{p}=\int Mult\left( \vec{k_{d}}\vert n_{d}, \vec{p} \right)Dir(\vec{p}\vert\vec{1}+ \vec{k_{s}})d\vec{p}= DirMult\left( \vec{k_{d}} \right\vert n_{d}, \vec{1}+ \vec{k_{s}})$ | ( 20 ) |
| --- | --- | --- |

The simulated and observed numbers of LGAs reporting at least one case in 3-month time bins are compared using a Poisson-binomial objective function. The probability of *k* LGAs (out of 44) reporting at least one case is equal to

|  | $\mathcal{L(}\vec{\theta})=P\left( k_{t} \vert\vec{p_{t}} \right)=\prod_{t} \left\{ \sum_{A \in F{}_{k}} \prod_{i \in A} p_{i, t}\prod_{j \in A^{c}} \left( 1-p_{j, t} \right) \right\}$ | ( 20 ) |
| --- | --- | --- |

where $F_{k}$ is the set of all subsets of *k* LGAs that can be selected from the 44 total LGAs. $p_{i, t}$ is the probability that LGA *i* reports at least one case in time period *t*: $p_{i, t}=1-\left( 1-R \right)^{I{}_{t}}$, where *R* is the case-to-infection ratio for a given simulation derived from the case count fit described about, and $I_{t}$ is the number of infections in time period *t*. This function is evaluated using the method described in [2]. As with the case counts, the total score is the product over time periods. Because this component of the objective function is more spatially granular than the statewide case counts, the time window was widened from 2 weeks in the case count fit to 3 months.

Finally, the objective function for the distribution of the LGA-level number of cases reported is treated as the probability of drawing the 44 observed case counts in time period *t* from an equally-weighted mixture of the 44 Poisson distributions from simulation:

|  | $F\left( x \right)= \sum_{i} Poiss(x\vert I_{i, t}, R)$ | ( 21 ) |
| --- | --- | --- |
|  | $\mathcal{L}\left( \vec{\theta} \right)=\prod_{t} \prod_{i} F(k_{i, t})$ | ( 22 ) |

Where $k_{i, t}$ is the observed case count in LGA *i* during time period *t.* The use of a mixture distribution here and the Poisson-binomial above, prevent explicit one-to-one mapping of LGAs in simulation and data, and instead aim to calibrate the simulation parameters to reproduce the general LGA-level characteristics of the data. This choice was made in part to reduce the space of calibration parameters; if simulated LGAs were explicitly mapped to their real counterparts, then an appropriate calibration space would include LGA-level parameters (particularly, campaign coverages). This re-parametrization would result in a large number of attendant calibration dimensions (43 additional dimensions for each parameter that is promoted from a statewide parameter to a set of LGA-specific parameters), which would require an infeasibly large number of simulations to appropriately sample the calibration space. Foregoing this one-to-one mapping in simulation and data allows LGA-level heterogeneity in campaign coverage to be more coarsely captured within a statewide mean and a coefficient of variation.

The total log-score visualized in plots throughout the main text is the sum of the logs of the first 2 (for single-node simulations) or all 4 (multi-node) components described above. To prevent a single component of the objective function from dominating the resampling, each component of the objective function is normalized by the number of data points used to compute that score.

**Overdispersion**

The choice of a Poisson approximation to a binomial objective function for fitting the case counts vs. time (described above) implies a particular mean-variance relationship. In practice, overdispersion may arise in both the disease transmission process and in the observation process. To test for the presence of overdispersion, a negative binomial objective function is compared with the Poisson objective function employed in this study. Only the simulations run to calibrate the final version of the model described in the manuscript are tested. For ease of interpretation, the negative binomial is specified by a probability *p* and an overdispersion parameter *r*, such that

|  | $Var\left( C_{t} \vert I_{t}, p, r \right)=pI_{t}\left( 1+\frac{pI_{t}}{r} \right)$ | ( 24 ) |
| --- | --- | --- |

The Poisson objective function is recovered for $r\to\infty.$ First, *r* is estimated separately for each calibration simulation. Figure 1 presents the distribution of these simulation-specific estimated dispersion parameters vs. the negative binomial log-score.


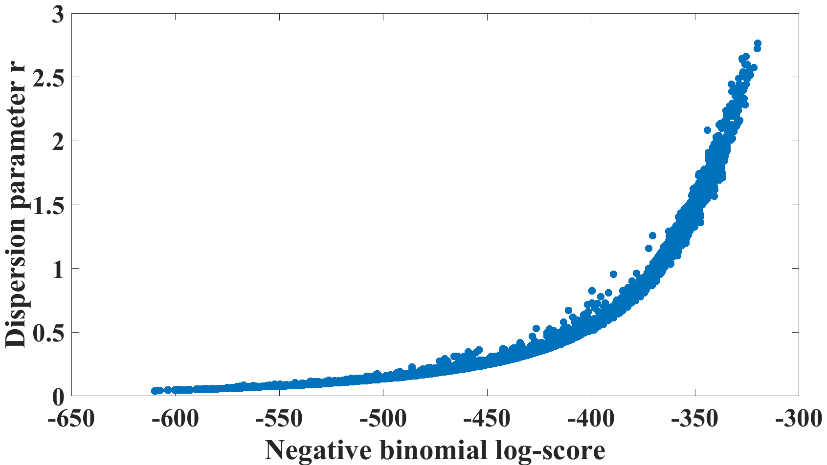


Figure 1: Distribution of the estimated dispersion parameter *r* vs. the negative binomial log-score of each calibration simulation.

It is clear that this estimated overdispersion is non-negligible. Two questions are critical. First, what proportion of excess variance is due to suboptimal model parameters and what is due to more fundamental sources in the dynamics and observation processes? Second, how does this affect the primary result of this work, the estimated probability of elimination vs. the time since the last observed paralysis case?

Without re-running the entire simulation suite with additional parameters to model excess dispersion in the dynamics and the observation process, it is not immediately obvious to the authors how to formally separate “parameter-based” overdispersion (i.e., large amounts of estimated overdispersion due to poor model parameters) from these more fundamental sources of overdispersion. The estimated dispersion parameters of the highest-scoring parameter sets are approximately 2.7, and this value is taken to represent some fundamental excess dispersion that cannot be eliminated through better model parameters. The scores and weights for the simulations are re-computed using a negative binomial objective function with the dispersion parameter *r* set to this value, and compared against the original Poisson weights in Figure 2. Comparing the ranges of the x and y axes reveals that, as expected, the inclusion of additional dispersion somewhat flattens the posterior weight distribution.


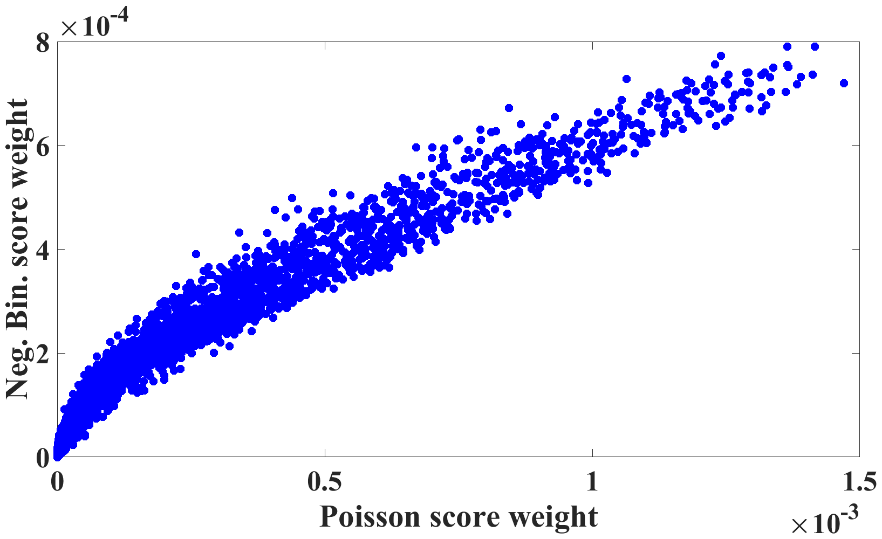


Figure 2: Calibration simulation weights computed using a Poisson objective function (x) and a negative binomial objective function (y) with dispersion parameter = 2.7. Comparing the scales shows that, as expected, allowing additional dispersion flattens the posterior weights.

What does this imply for the distribution of probability of eradication vs. time since last observed paralysis case? To investigate this, the simulations are re-weighted and the distribution is recomputed, according to Eqn. ( 25 ):

|  | $P{}_{erad}\left( t \right)= \frac{\sum_{\{sims \vert I\left( t \right)=0\}} Bin\left( 0 \vert I_{c}\left( t \right), .005 \right)*\frac{w_{NB}}{w_{P}}}{\sum_{\{all sims\}} Bin(0\vert I_{c}\left( t \right), .005)*\frac{w_{NB}}{w_{P}}}$ | ( 25 ) |
| --- | --- | --- |

Where $w_{NB}$ and $w_{P}$ represent the negative binomial and Poisson weights, respectively, of a given simulation, and all other terms are defined as in the main text, Results and Discussion section. A complication is worth noting; while this reweighting scheme ought to work in principle, a complication arose in practice. The reweighting factor $\frac{w_{NB}}{w_{P}}$ implicitly assumes that the simulation parameter sample distribution is proportional to the posterior weights $w_{P}$. The initial round of the calibration procedure uniformly samples the parameter space, and subsequent rounds resample proportionally to the estimated posterior weights. Some of these initial samples provide exceedingly poor fits to the data, with weights many orders of magnitude below the peak weights; unless enormous numbers of simulations are run, these simulations are over-represented in the final sample. The reweighting factors $\frac{w_{NB}}{w_{P}}$for these samples are also quite large (allowing for overdispersion improves the score of simulations that fit poorly in the mean much more than it improves those that already fit well). To prevent these from dominating the reweighted curve, a cut is placed so that the sums extend over only simulations with weights within five orders of magnitude of the highest weights.

Figure 3 compares the original and reweighted curves of probability of eradication given time since last observed paralysis case. The curve shifts only minimally with the reweighting. A couple of potential reasons for the similarity: because parameter sets were preferentially sampled from the high-score region of parameter space, this reweighting factor is not very far from 1 for most simulations (e.g., roughly 1/2 of all reweighting factors lie between 0.5 and 3). Furthermore, the near-eradication dynamics may not be substantially different in the simulations that received higher weights and those that received lower weights in the reweighting, preserving the overall shape of this curve.
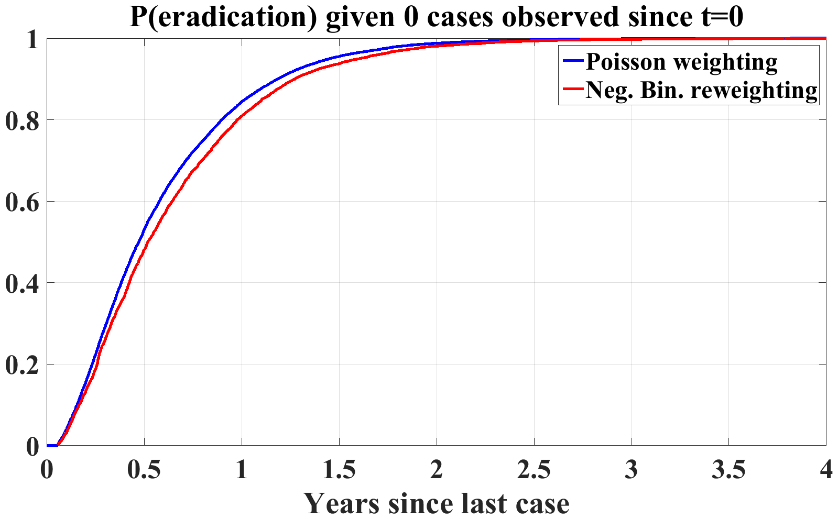


Figure 3: Comparison of the probability of eradication vs. time since last case for Poisson simulation weights (blue, as in main manuscript) and with negative binomial weights (red). The distribution remains largely similar despite reweighting of the individual simulation runs.

**Metapopulation model construction**

The spatial metapopulations, henceforth referred to as nodes, are constructed beginning with WorldPop [ref] population maps. The 2014 population map is provided as grid cells with a spatial resolution of 3x3 arcseconds. The representation of Kano state in this basis contains approximately 2.4 million population cells, making this representation unwieldy if each grid cell represents a node in the model. Furthermore, the distribution of population is highly clustered, with a majority (~98%) of grid cells having estimated populations below 10 individuals; the remaining 2%, about half contain populations from 10-100, and the other half ranges from 100-1000. Representing each grid cell as a node is thus also an inefficient representation for modeling purposes, and due to the spatially clustered nature of the population, this is not alleviated by simply summing adjacent cells to decrease the grid resolution.

The following method was employed to aggregate WorldPop grid cells into a reduced set of spatially clustered populations, which are assumed to be internally well-mixed. The LGA of Kabo within Kano state will be used to illustrate the steps of the algorithm. The first step in this method is to identify high-population grid cells. There are a number of possible definitions of “high-population”; in the case of Kano state, there exists a clear gap in the distribution of population per grid cell at a value 10/cell (Figure 1), this value is thus chosen as a simple cut to separate high-population and low-population grid cells.

Spatially contiguous groups of high-population grid cells are then identified. Figure 2(a) shows the population image of Kabo LGA. The population raster is first converted into a raster of logical 1’s and 0’s by the cut at 10 people/grid cell (Figure 2(b)). A binary closing operation is performed with a 4-way structuring element; this operation unifies nearby features that are separated by a single low-population pixel (Figure 2(c)); in the image, the two population features just southwest of center are unified. High-population components that are connected with a 4-way structuring element are then found and labeled (Figure 2(d)). Each labeled island is aggregated into a single node by summing the population of the component cells, and placing the location at the population-weighted centroid of the island.

The next step involves handling the remaining “background” population. The population centers identified in the previous step are used as the generator set for a Voronoi tessellation of the population map. All population within a particular Voronoi cell is assigned to the associated generator point (Figure 2(e)). The full population of the original map is preserved and represented by a reduced set of population centers, with the implicit assumption that the background population is well-mixed with the nearest population center. When the shape of the state or district being partitioned is non-convex, this method can produce node that are non-contiguous and cross concave boundaries. In the application presented in this paper, this issue was found to occur infrequently, and resulted in the mis-assignment of sufficiently small populations that it was allowed. For a more robust implementation, the problem could be avoided entirely by tessellating the space based on geodesic distance within the administrative boundary rather than on a simple Euclidean distance between each pair of points [refer to occlusion point geodesic paper, k-voronoi diagrams paper].

Finally, nodes are merged based on two criteria – any node with populations below 1000 is merged with its nearest neighbor, and any pair of nodes that are less than 1.5 km from each other are also merged. The above algorithm is applied separately to each of the 44 LGAs in Kano state, so that LGA populations are preserved and each node can be unambiguously mapped to a single LGA for comparison with data. The 2.4 million grid cells in the original image (Figure 3(a)) are reduced to a set of 1300 nodes (Figure 3(b)), with populations ranging from 1k to 500k individuals.


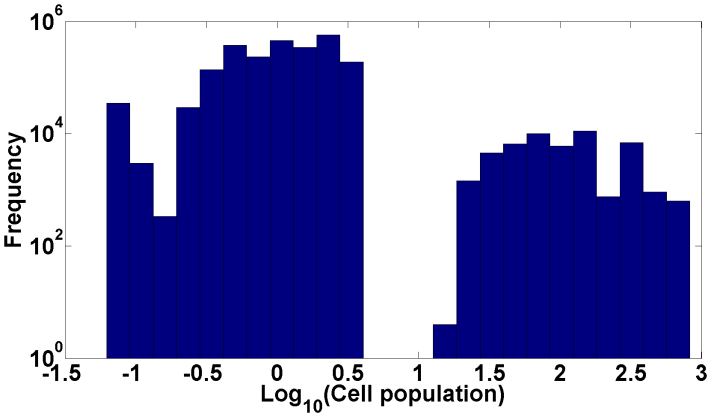


Figure 4: Distribution of population per grid cell (3x3 arcseconds) in Kano state. Source data from Worldpop[3], 2010, adjusted to match UN national estimates, version 4.


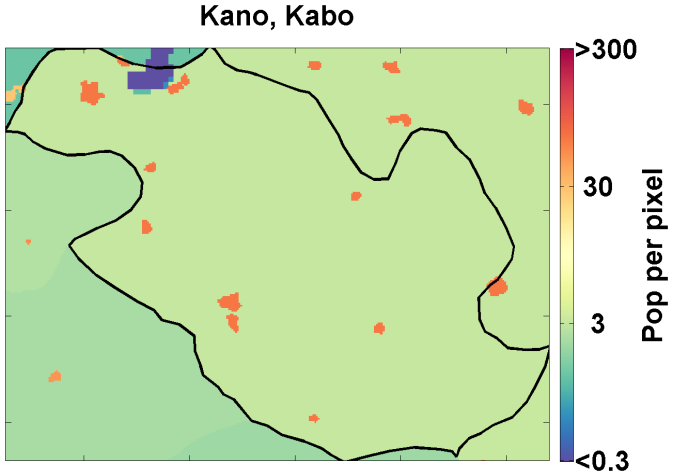

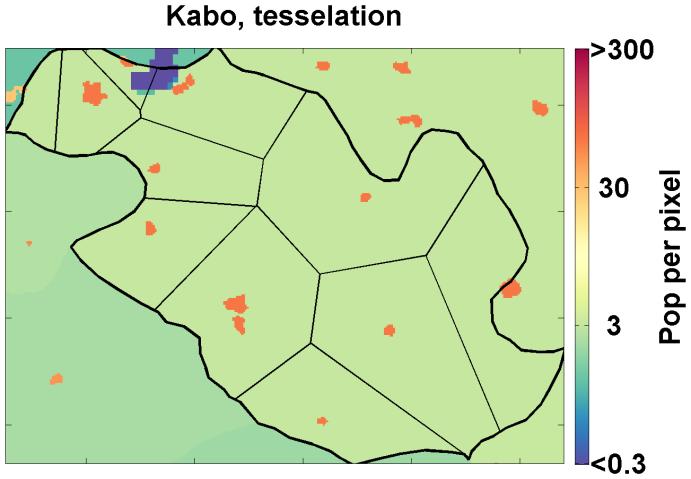

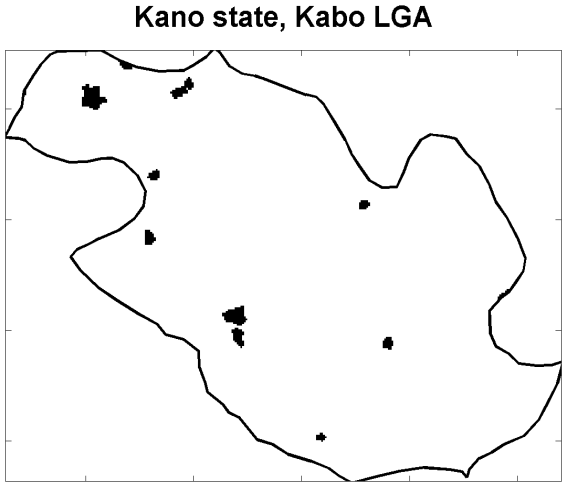

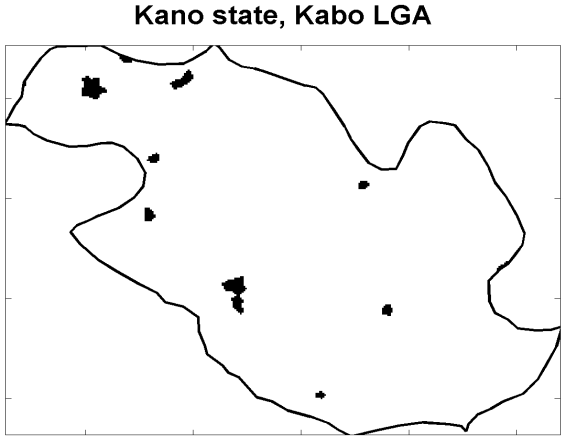

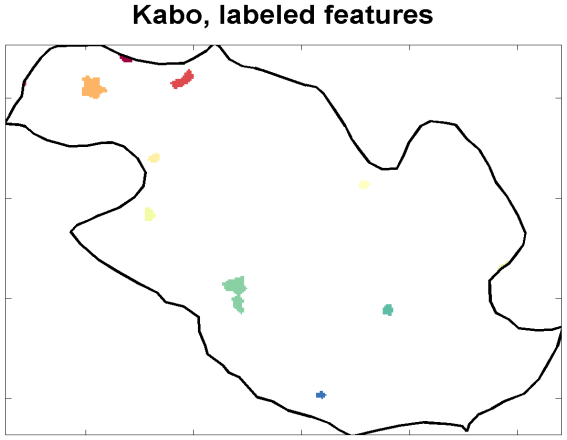


Figure 5: (a) Population distribution in Kabo LGA, Kano. Source Worldpop. (b) Application of cut at 10 people/grid cell. (c) Application of binary closing operation. Comparing to (b), the major change is that the two population features just southwest of center have been joined. (d) Labeling of connected features. Each color represents a different island. (e) Voronoi tessellation of the LGA, with features identified in (d) used as the generating points.


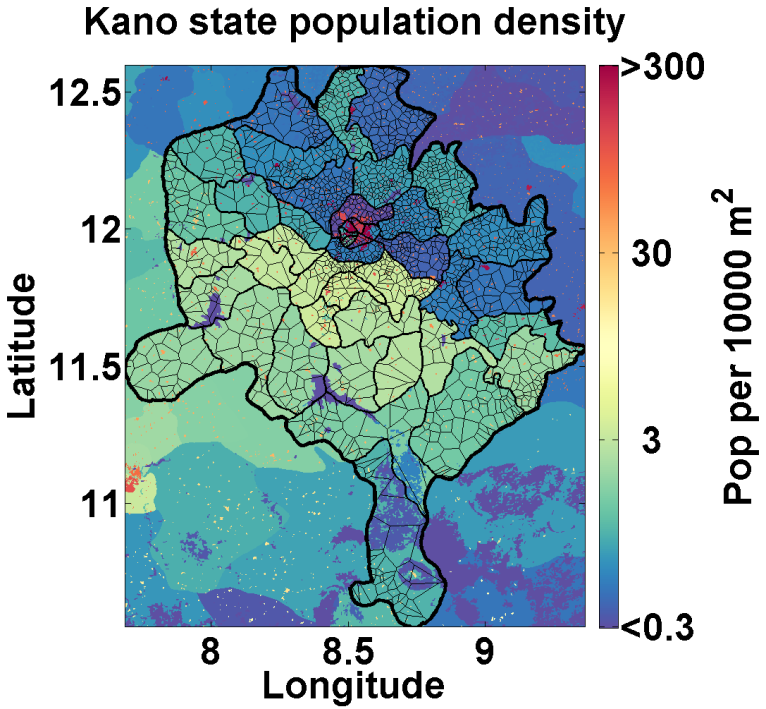

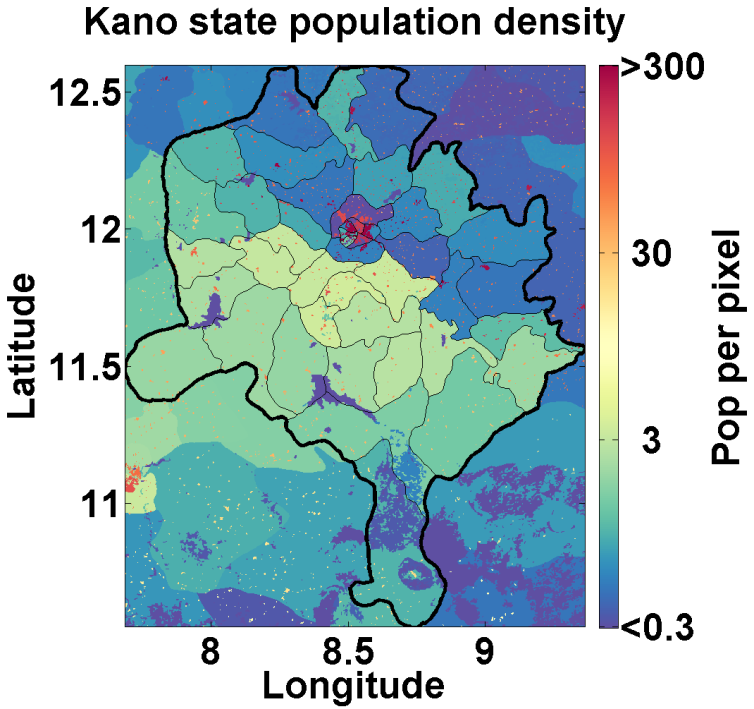


Figure 6: (a) Population distribution, Kano State, Nigeria. Thick line outlines Kano state, thin lines outline the 44 LGAs. Source Worldpop. (b) Final nodeset used for simulation. Each thinly outlined polygon represents a single aggregated metapopulation in the model.

[1] N. P. C.- NPC/Nigeria and I. International, “Nigeria Demographic and Health Survey 2013.” 2014.

[2] M. Fernandez and S. Williams, “Closed-Form Expression for the Poisson-Binomial Probability Density Function,” *IEEE Trans. Aerosp. Electron. Syst.*, vol. 46, no. 2, pp. 803–817, Apr. 2010.

[3] “Worldpop - Selected Data: Africa > Nigeria > Population.” [Online]. Available: http://www.worldpop.org.uk/data/summary/?contselect=Africa&countselect=Nigeria&typeselect=Population. [Accessed: 17-Sep-2015].
